# Hypnotics use in children 0–18 months: moderate agreement between mother-reported survey data and prescription registry data

**DOI:** 10.1186/s40545-017-0117-7

**Published:** 2017-09-08

**Authors:** Ingvild Holdø, Jørgen G. Bramness, Marte Handal, Leila Torgersen, Ted Reichborn-Kjennerud, Eivind Ystrøm, Hedvig Nordeng, Svetlana Skurtveit

**Affiliations:** 1 Norwegian Centre of Addiction Research (SERAF), University of Oslo, Oslo, Norway; 2 Norwegian National Advisory Unit on Concurrent Substance Abuse and Mental Health Disorders, Ottestad, Norway; 3 Domain of Mental and Physical health, Norwegian Institute of Public Health, Oslo, Norway; 4 Institute of Clinical Medicine, University of Oslo, Oslo, Norway; 5 PharmacoEpidemiology and Drug Safety Research Group, School of Pharmacy, University of Oslo, Oslo, Norway; 6 Section of Health, Developmental and Personality Psychology, Department of Psychology, University of Oslo, Oslo, Norway

**Keywords:** MoBa, Hypnotics and sedatives, Agreement, Child, Prescriptions

## Abstract

**Background:**

Different methods in pharmacoepidemiology can be used to study hypnotic use in children. But neither questionnaire-based data nor prescription records can be considered a “gold standard”. This study aimed to investigate the agreement between mother-reported questionnaire-based data and prescription record data for hypnotic drugs in children aged 0–18 months. The agreement was compared to the agreement for a group of antiepileptic drugs.

**Methods:**

Prescription record data were collected from the Norwegian prescription database for 47,413 children also surveyed in the Norwegian mother and child cohort between 2005 and 2009. Agreement between in the two data sources was calculated using Cohens Kappa. Multinomial logistic regression was used to calculate the effect of sociodemographic variables on discrepancies in data sources.

**Results:**

The agreement between mother-reported and dispensed hypnotics was less than 50% for all hypnotics. Sensitivity of reporting increased with number of filled prescriptions. The agreement of antiepileptic drugs was 92.9% in the same population. Of several sociodemographic factors only paternal educational level and maternal work situation was significantly related to agreement between prescription record and survey data.

**Conclusion:**

There was a moderate agreement between reported use and dispensed hypnotic drugs for infants and toddlers. Results indicate that sociodemographic factors play only a minor role in explaining discrepancy.

**Electronic supplementary material:**

The online version of this article (10.1186/s40545-017-0117-7) contains supplementary material, which is available to authorized users.

## Background

Prescribing sleep-inducing medication for otherwise healthy infants and toddlers is a controversial subject. Different countries have different therapeutic traditions, but pharmacological treatment of sleep problems in the youngest children is frequent in many countries [1–4]. For infants and toddlers antihistamines, α_2_-agonists or chloral hydrate are most often the drugs of choice, while melatonin, benzodiazepines and the benzodiazepine like z-hypnotics (i.e. zolpidem, zopiclone) are mainly prescribed for older children and teen-agers [3, 4]. In Norway, the antihistamine alimemazine (an aliphatic phenothiazine) is the most used sleep inducing drug in infants and toddlers, despite not being approved for the age group less than 2 years of age [1, 3, 5–9].

There is a lack of evidence of effect and safety for sleep inducing drugs to small children. This is especially true for the older drugs introduced in a time where drugs testing was less regulated. To compensate for this, observational studies of real life use in children can be useful. There are many types of data sources for such studies and all will have their strengths and limitations, but few studies have estimated the quality of different data sources on the use of prescription drugs in young children. Two such data sources may be survey data and prescription data. However both sources have their limitations and none of them can be considered the “gold standard” [10]. One prior study, comparing the Norwegian Prescription Database (NorPD) with maternal report of prescribed anti-asthma drugs for children aged 7 years in the Norwegian Mother and child cohort study (MoBa) showed high agreement with maternal report [11]. However, little is known about other drugs.

The aim of this study was to investigate the agreement between maternal-report on sleep inducing drug use by 0–18 month’s old children in the Norwegian Mother and Child Cohort with information on dispensed hypnotic drugs from pharmacies in the Norwegian Prescription Database. The results were compared with the agreement for antiepileptic drugs that should be taken more regularly for a chronic somatic disease, thus theoretically representing a drug with an expected higher agreement between the two data sources. Since alimemazine traditionally is used in Norway as a hypnotic drug to young children, we finally estimated the extent to which sociodemographic background variables and the number of prescriptions of alimemazine could account for possible discrepancies.

## Methods

### MoBa

The Norwegian Mother and Child cohort study (MoBa) is a prospective population-based pregnancy cohort study conducted by the Norwegian Institute of Public Health and aims to study causes of disease [12, 13]. Women were invited to join after attending routine ultrasound appointment in pregnancy week 18. From one participating hospital in one region in 1999 the study gradually included more hospitals. In the period 2005–2008 almost all Norwegian women were invited to participate. Overall in the period 1999 to 2008 approximately 41% of the invited consented to participation. The last birth into the cohort was in June 2009. The MoBa study collected questionnaires at specific time points; our study uses questionnaires from baseline and at child’s age 6 and 18 months [14]. The personal identification numbers [15] assigned to all individual in Norway allow for linking of MoBa data with other registries and studies. In this study, MoBa data was linked to data from the Norwegian Prescription Database.

### Norwegian prescription record (NorPD)

NorPD holds a complete record of all dispensed prescription drugs since 2004 [16]. Pharmacies are obliged to register prescriptions electronically and submit information every month. NorPD includes both prescriptions made to individuals and institutions, but prescriptions in institutions are not registered at a person level [17]. For each prescription drug specific information, patient data and prescriber information are registered. In the present study we only studied prescriptions made to children in the MoBa study. We used information on patient ID, drug name, date of dispensing, and reimbursement code.

### Sample

**Fig. 1:**
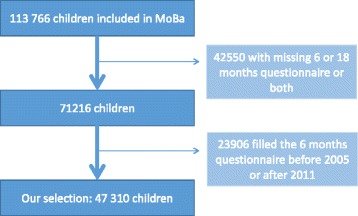
Inclusion in study based on participants in the Norwegian Mother and child cohort study

To ensure that information was available from both data sources, we only included children in MoBa when the 6 month questionnaire was filled after 1. January 2005. Both the 6 months and the 18 months questionnaire had to be filled in for the child to be included in our study. The study population thus consisted of 47,310 children (see Fig. 1).

From baseline questionnaires we used the following information: parents’ age, family income, parents’ education, if the pregnancy was planned or not, maternal marital status, maternal parity and maternal employment status. From the 6 and 18 months questionnaires we used reported drug use (Additional file 1). Trained personnel interpreted and converted drug names from the questionnaires to ATC codes when entering data.

### Drugs included in the study

For the present study information on all anxiolytics, hypnotics and sedatives, dexchlorpheniramine, alimemazine and promethazine was used (Table 1). As comparison we also included a group of antiepileptic drugs recommended as first choices for treatment of childhood epilepsy [18]; Carbamazepine, oxcarbazepine, lamotrigine, topiramate, and valproate.

**Table 1:** Drug name and number of prescriptions for all sedative drugs from birth until 18 months of age^a^ in our selection of children in the MoBa cohort, 3071 children with unique IDs

Drug name	Atc code	N° of prescriptions	Main reimbursed diagnosis	Reimbursement (%)
clonazepam	N03AE01	23	epilepsy	69.57%
diazepam	N05BA01	399	epilepsy	14.79%
clobazam	N05BA09	7	epilepsy	42.86%
hydroxyzine	N05BB01	3	allergy	66.67%
nitrazepam	N05CD02	41	epilepsy	68.29%
midazolam	N05CD08	14	epilepsy	57.14%
melatonin	N05CH01	33	(no reimbursement)	0.00%
deksklopheniramin	R06AB02	3038	allergy	42.59%
alimemazine	R06AD01	993	allergy	2.72%
sum		4551		31.58%

^a^Date of filling of 18 months questionnaire used as cut off

From NorPD we included hypnotics and antiepileptics dispensed to the child from birth until the date the mother filled the 18 months questionnaire.

### Analysis strategy and statistics

- Reported (yes/no) and dispensed prescriptions (yes/no) for all hypnotic drugs and the control group of antiepileptic drugs were cross-tabulated. We used Cohen’s Kappa with Fleiss’ subdivision to determine agreement between reported use and registry data (Table 1) [19].
- Prescriptions for alimemazine were subdivided into categories based on number of prescriptions (0, 1, 2 and 3 or more) and we compared this to information on reported alimemazine use from the questionnaires(yes/no). Setting prescription record data as reference standard, we measured sensitivity of reporting by number of prescriptions (Table 2).
- Mother-reported sociodemographic data was used to characterize the children with discrepancy between reported use and dispensed prescriptions of alimemazine. We compared three groups: 1) Only mother-reported alimemazine use in MoBa, 2) Only dispensed alimemazine in the NorPD prescription record and 3) Both mother-reported use and dispensed prescriptions for alimemazine. The groups were compared using the Chi-square test (Table 3). The sociodemographic variables showing significance (*p* < 0.1) in Table 3 were included in a multinomial logistic regression analysis. The outcome measure was the relative risk (RR) with confidence intervals, using the category both mother-reported use and dispensed prescriptions for alimemazine as reference category.

**Table 2:** Agreement between at least one-time mother reported use of hypnotics and at least one dispensed prescription for a hypnotic drug to children aged 0–18 months

	Maternal report (MoBa)			Dispensed drugs (NorPD)			Neither	Only MoBa	Only NorPD	Both		Agreement^d^
Drug category (ATC code)	n	%	95% CI	n	%	95% CI	n	n	n	n	total	
Alimemazine (R06AD01)	367	0.78%	0.70–0.86%	745	1.58%	1.47–1.69%	46,459	106	484	261	47,310	46.3
Promethazine (R06AD02)	14	0.03%	0.02–0.05%	0			47,297	14	0	0	47,310	0
Dexchlorpheniramine (R06AB02)	1131	2.39%	2.26–2.53%	2101	4.44%	4.26–4.64%	44,811	398	1368	733	47,310	43.6
Hypnotics and sedatives (N05C)^a^	39	0.08%	0.06–0.11%	38	0.08%	0.06–0.11%	47,249	23	22	16	47,310	41.0
Anxiolytics (N05B)^b^	62	0.14%	0.11–0.17%	351	0.74%	0.67–0.82%	46,935	24	311	40	47,310	19.1
Recommended antiepileptic’s (from ATC N03A)^c^	49	0.10%	0.08–0.14%	50	0.11%	0.08–0.14%	47,257	3	4	46	47,310	92.9

^a^includes nitrazepam, midazolam and melatonin

^b^includes diazepam, clobazam and hydroxyzine

^c^First choice drugs for treatment of childhood epilepsy. Includes carbamazepine N03AF01, oxcarbazepine N03AF02, lamotrigine N03AX09, topiramat N03AX11, and valproate N03AG01. Recommended by the Norwegian Society of Paediatricians

^d^Calculated using Cohen’s Kappa

**Table 3:** Validity of maternal report on use of compared to number of dispensed prescriptions of alimemazine

	Neither	MoBa	NorPD	Both	
Alimemazine R06AD01	*n*	*n*	*n*	*n*	Sensitivity^b^
Total^a^	46,462	106	485	261	34.99%
1 prescription	–	–	415	180	30.25%
2 prescriptions	–	–	55	51	48.11%
3 or more prescriptions	–	–	15	30	66.67%

^a^total number of children included is 47,314

^b^dispensed prescriptions is reference standard for sensitivity analysis

Statistical analyses were conducted using SPSS version 22 for Windows.

### Ethics

Informed consent was obtained from mothers before inclusion in MoBa. The Regional Committees for Medical and Health Research Ethics and the Norwegian Data Protection Authority approved the linkage of NorPD and MoBa.

## Results

### Prevalence of hypnotic use

The prevalence of hypnotic drug use was lower in the mother-reported MoBa study than in the prescription database NorPD for all groups of medications except promethazine (Table 2). Alimemazine and dexchlorpheniramine were the two substances most frequently reported used and dispensed. Alimemazine was reported used by 367 (0.8%) children, but was dispensed to 746 (1.6%) children under the age of 18 months. Dexchlorpheniramine was reported used by 1131 (2.4%) children in MoBa, but 2103 (4.4%) had filled a prescription according to NorPD.

### Agreement between mother-reported use (MoBa) and dispensed (NorPD) hypnotic drugs.

The agreement between the two data sources was lower than 50% for all the hypnotic drugs. It was best for alimemazine (46.3%) and poorest for anxiolytics (19.1%). The agreement was, however, excellent for the group of antiepileptic’s recommended for childhood epilepsy (92.9%) (Table 2).

The sensitivity of the mother-reported alimemazine use increased by numbers of prescriptions filled. Sensitivity was 30.3% when the children had received only one prescription, increasing to 48.1% for children receiving two prescriptions and to 66.7% for those receiving 3 prescriptions or more (Table 3).

### Sociodemographic background variables

The differences of sociodemographic characteristics between mother-reported use and prescription record data of alimemazine use are shown in Table 4. The adjusted multinomial logistic regression analysis showed that when fathers had higher education the risk of children’s alimemazine use being only mother-reported was 2.3 times higher (95% Confidence interval [95% CI] 1.3–3.9) than having consistent data in both mother-reported MoBa and prescription record NorPD. Mothers being unemployed increased the risk of children being in the prescription record group only, 1.9 times (95%CI 1.0–3.4) as opposed to the group with consistent data in both sources(data not shown).

**Table 4:** Sociodemographic characteristics of alimemazine use categorized in three groups: reported use in MoBa but no prescriptions in NorPD, no reported use in MoBa but prescription in NorPD and reported use in MoBa and prescription in NorPD

		Only in MoBa^a^ (*N* = 106)		Only in NorPD^b^(*N* = 485)		Both MoBa & NorPD^c^ (*N* = 261)		*P*-value****
Socioeconomic variables		n	%	n	%	n	%	
Mothers education (*n* = 822)	low (≤12)	30	29.41%	170	36.48%	106	41.73%	
	high (≥13)	72	70.59%	296	63.52%	148	58.27%	0.083
Fathers education (*n* = 766)	low (≤ 12)	35	38.46%	245	56.71%	145	59.67%	
	high (≥13)	56	61.54%	187	43.29%	98	40.33%	0.002
Planned pregnancy (*n* = 837)	no	17	16.50%	101	21.26%	48	18.53%	
	yes	86	83.50%	374	78.74%	211	81.47%	0.448
Maternal employment situation (*N* = 839)	not working	4	3.88%	53	11.13%	17	6.56%	
	working	99	96.12%	423	88.87%	242	93.44%	0.019
Family income (*N* = 818)	low	22	21.57%	126	27.21%	72	28.46%	
	medium	64	62.75%	288	62.20%	161	63.64%	
	high	16	15.69%	49	10.58%	20	7.91%	0.227
Maternal marital status (*N* = 851)	married/cohabiting	101	95.28%	471	97.31%	249	95.40%	
	other	5	4.72%	13	2.69%	12	4.60%	0.312
Parity (*N* = 851)	0	44	41.51%	211	43.60%	122	46.74%	
	1	47	44.34%	168	34.71%	83	31.80%	
	≥2	15	14.15%	105	21.69%	56	21.46%	0.164
Age mother (years) (N = 851)	≤29	41	38.68%	185	38.22%	122	46.74%	
	≥30	65	61.32%	299	61.78%	139	53.26%	0.069
Age father (years) (N = 851)	≤29	26	24.53%	126	26.03%	77	29.50%	
	≥30	80	75.47%	358	73.97%	184	70.50%	0.5

****Chi-square test

^a^Only reported use in MoBa. No prescription in NorPD

^b^Only filled prescription in NorPD. No reported use in MoBa

^c^Both reported use in MoBa and filled prescription in NorPD

## Discussion

The agreement between mother-reported hypnotic drug use by their child and data on dispensed prescriptions was found to be moderate to poor for hypnotic drugs. For alimemazine, agreement increased with increasing numbers of dispensed prescriptions. With the exception of paternal education and mothers work situation, no differences in social factors between reported use and dispensed alimemazine were seen.

Mother-reported drug use was generally lower than prescriptions would imply. Some of the dispensed drugs were probably not taken as the questionnaires asked for *use* of medication not received prescriptions. Theoretically, using filled prescriptions as opposed to prescribed prescriptions should increase the adherence, as we at least know that the drugs were in the patient’s possession. However, when using data on dispensed medication we still do not know if the medication was actually taken or subject to so called secondary non-compliance. Many patients are not adherent to prescribed drug therapy [20]. A Canadian study of non-adherence found that sedative- hypnotics had a 35% frequency of non-adherence among the general population [21]. The lower frequency of mother-reported drug use could in this sense be closer to reality than dispensed prescriptions.

Reporting -no use of a hypnotic drug even when a prescription had been filled can also be due to lack of memory. It’s more difficult to remember drug use happening a long time ago [10, 22], drugs taken as needed [23–25] and drug use when asked open-ended questions [26]. Hypnotic drugs for small children is controversial and thus might be afflicted with feelings of shame and guilt by the mother, which in turn could have caused lower agreement. Others studies have shown larger discrepancies between prescribed and reported use when the drugs are afflicted with stigma [24, 27]. In MoBa the mothers were asked open questions about their child’s drug use up to a year earlier. On the other hand, the high agreement we found for chronically used antiepileptic drugs indicates that the long time periods and open-ended questions was not a problem. A study validating the same data sources in children aged 7 years but with specific asthma questions found higher agreement [11]. The agreements we found for systemic antihistamines and for antiepileptic drugs were similar to those found among pregnant women reporting on their own use of drugs in an Italian study [28].

Perhaps more surprising is the large number of mothers who reported use of alimemazine even if no prescription was filled. Possible explanations could be that the drugs had been prescribed for someone else, purchased abroad or even been given to the child while in hospital, such use would not be captured in the prescription database. Alimemazine is frequently used as sleep inducer for children in hospitals [16].

A large proportion of prescriptions to the age group 0–2 years had missing identification numbers in the years 2006–2007 due to a problem with assignment of these numbers to new-borns (personal communication Olaug Sveinsgjerd Fenne). However, the high agreement for antiepiletics might indicate that this problem was not dominant in our selection.

We did not find any substantial systematic differences of sociodemographic variables on discrepancy between mother-reported information and data on dispensed medication. Family income, marital status, parity, mothers age, if the pregnancy was planned or not and mother’s education were all similar between the groups. Non-working mothers had a higher percentage of drug only reported in the prescription record whereas families with college educated fathers had more use only reported in the cohort study. But considering that other similar variables like family income and maternal education level did not influence the outcome this might not be clinically relevant findings. Results from other studies indicate that the effect of sociodemographic variables on recall is conflicting. Some find no effect of sociodemographic variables [29, 30] whereas age and education level have been shown to affect recall, but in different directions [10, 28, 31–34].

The Norwegian Mother and Child cohort study included more than 100,000 pregnancies and continues to follow the children through life. Of the included women 87% completed the 6 months questionnaire and 77% completed the 18 months questionnaire [35]. Compared with the average pregnant woman the participants were more often living with a partner, less likely to smoke, more likely to be compliant to health advice, older and less likely to have more than two children, despite of this the bivariate associations between exposure and outcome variables for eight different conditions were not statistically different from those of the general population [12, 35]. In MoBa mothers are asked to remember drugs use up to 1 year prior. Large longitudinal studies like MoBa must make a trade-off between amount of information and the burden placed on the participants. The scope of this study was to evaluate existing sources of drug use information with the limitation they have.

This study is one of very few studies comparing mother-reported survey data with prescription data for hypnotic use among infants and toddlers. Reliable sources of information on medication use are important in clinical and epidemiological research. In the paediatric field there are still many unanswered questions about drug use and drug safety and evaluating data sources in this field is thus especially interesting. Like former studies performed on adults and adolescents this study on children shows that generally agreement between different sources of drug use is better for prescription drugs used chronic than drugs taken as needed [36, 37].

## Conclusion

There was a moderate agreement between reported use and dispensed hypnotic drugs for infants and toddlers. There were, however, few differences between the two sources of data when considering sociodemographic variables. This might indicate that there are not systematic differences between the group who has been dispensed an hypnotic drug and those who report to have used it.

## Additional file

Additional file 1: Shows the questions concerning medication use from the Norwegian Mother and Child cohort study questionnaire Q4 (6 months of age) and Q5 (18 months of age). (DOCX 971 kb)

## Abbreviations

MoBa: Norwegian mother and child cohort study
NorPD: Norwegian prescription database
