# Supplementary material for: Hypnotics use in children 0–18 months: moderate agreement between mother-reported survey data and prescription registry data
Source: J Pharm Policy Pract. 2017 Sep 8;10:28. doi: 10.1186/s40545-017-0117-7 (PMC5591515; doi:10.1186/s40545-017-0117-7)
Supplement: Additional file 1: — Shows the questions concerning medication use from the Norwegian Mother and Child cohort study questionnaire Q4 (6 months of age) and Q5 (18 months of age). (DOCX 971 kb) [file 40545_2017_117_MOESM1_ESM.docx]

**Additional file 1**

Shows the questions concerning medication use from the Norwegian Mother and Child cohort study questionnaire Q4 (6 months of age) and Q5 (18 months of age).


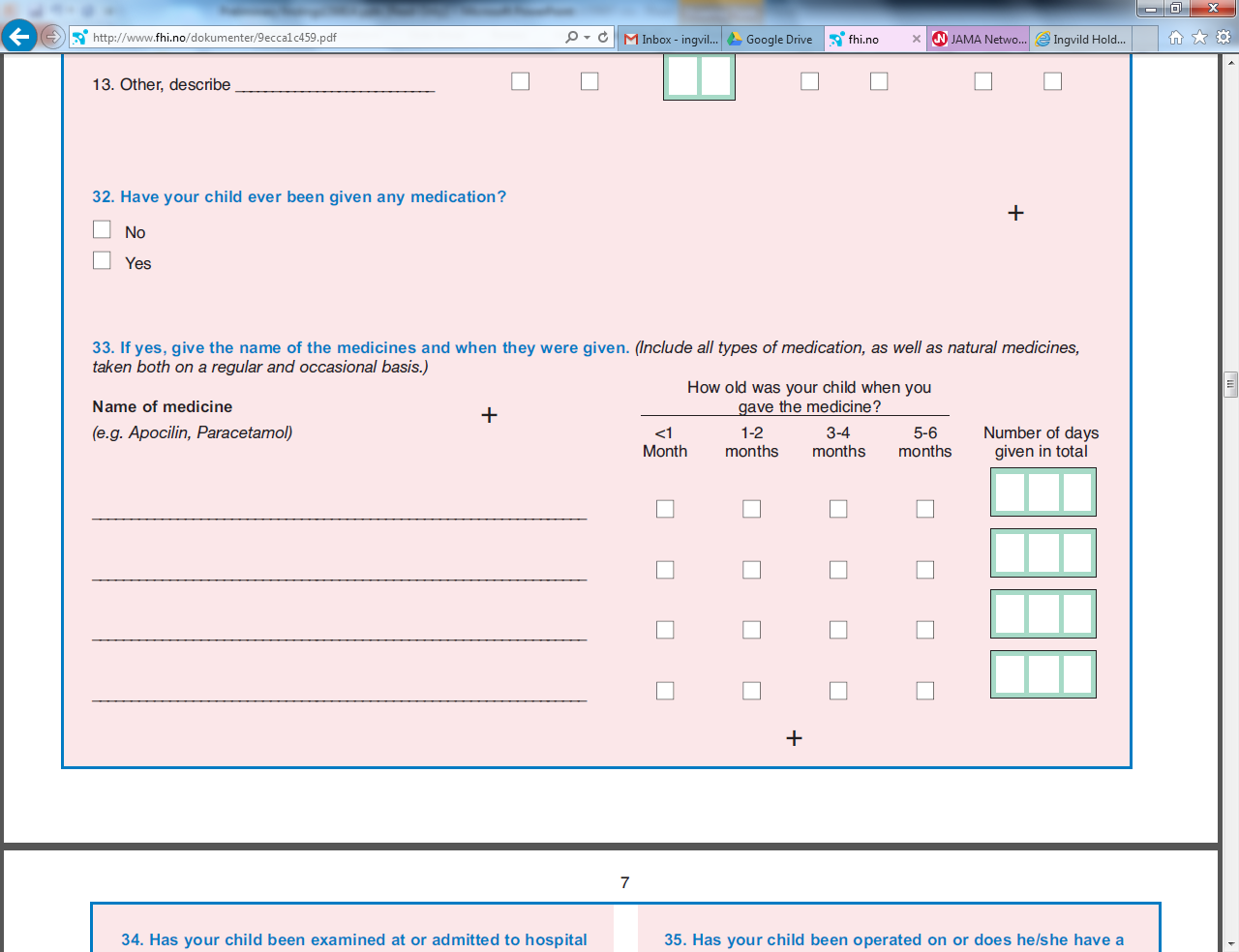


Medication questions from Q4 (6 months) questionnaire


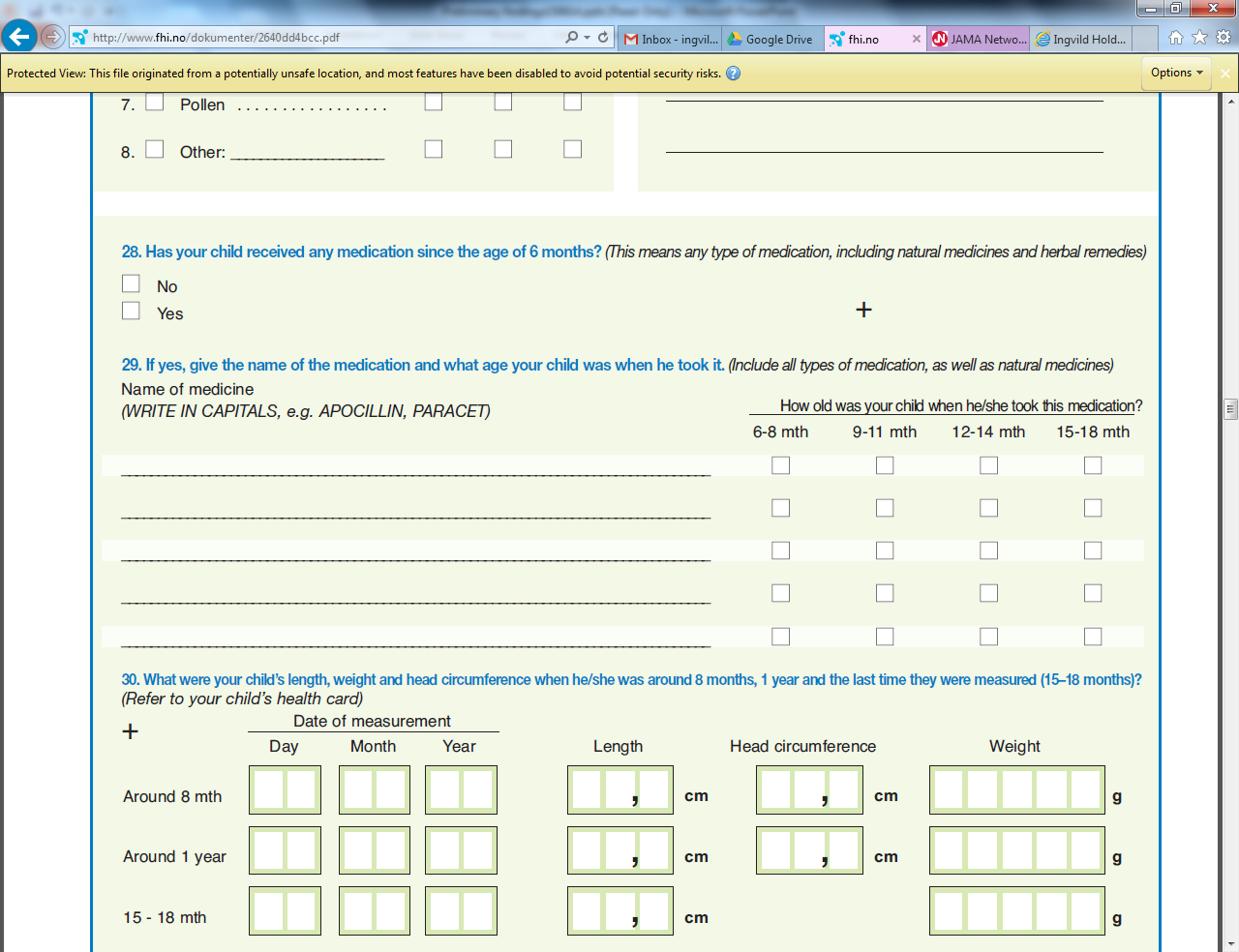


Medication questions from Q 5 (18 months) questionnaire
